# High Efficiency RNA Extraction From Sperm Cells Using Guanidinium Thiocyanate Supplemented With Tris(2-Carboxyethyl)Phosphine

**DOI:** 10.3389/fcell.2021.648274

**Published:** 2021-04-21

**Authors:** Martin Roszkowski, Isabelle M. Mansuy

**Affiliations:** Laboratory of Neuroepigenetics, Brain Research Institute, Medical Faculty of the University of Zurich, and Institute for Neuroscience, Department of Health Science and Technology of the ETH Zurich, Zurich, Switzerland

**Keywords:** sperm cell, RNA, lysis and extraction, mouse, disulfide bond, TCEP, guanidinium thiocyanate, pH

## Abstract

The extraction of high-quality ribonucleic acid (RNA) from tissues and cells is a key step in many biological assays. Guanidinium thiocyanate-phenol-chloroform (AGPC) is a widely used and efficient method to obtain pure RNA from most tissues and cells. However, it is not efficient with some cells like sperm cells because they are resistant to chaotropic lysis solutions containing guanidinium thiocyanate such as Buffer RLT+ and Trizol. Here, we show that disulfide bonds are responsible for the chemical resistance of sperm cells to RNA extraction reagents. We show that while β-mercaptoethanol (βME) can increase sperm lysis in Buffer RLT+, it has no effect in Trizol and leaves sperm cells intact. We measured the reduction of disulfide bonds in 2,2′-dithiodipyridine (DTDP) and observed that βME has a pH-dependent activity in chaotropic solutions, suggesting that pH is a limiting factor. We identified tris(2-carboxyethyl)phosphine (TCEP) as an efficient lysis enhancer of AGPC solutions that can retain reducing activity even at acidic pH. Trizol supplemented with TCEP allows the complete and rapid lysis of sperm cells, increasing RNA yield by 100-fold and resulting in RNA with optimal quality for reverse transcription and polymerase chain reaction. Our findings highlight the importance of efficient cell lysis and extraction of various macromolecules for bulk and single-cell assays, and can be applied to other lysis-resistant cells and vesicles, thereby optimizing the amount of required starting material and animals.

## Introduction

Ribonucleic acid (RNA) is a macromolecule essential for many biological processes across all known species. It exists in different forms and length, and has numerous functions. In eukaryotes, messenger RNA (mRNA) is a form of coding RNA that is transcribed from genes and serves as template for translation into proteins (Buccitelli and Selbach, 2020). Non-coding RNA is transcribed from intergenic regions, and is not translated into proteins but has various regulatory functions. MicroRNAs and long non-coding RNA are involved in the silencing/degradation of mRNA and in genome regulation, while ribosomal and transfer RNA participate to ribosomal constitution and functions, respectively (Holoch and Moazed, 2015; Quinn and Chang, 2016), and small interfering RNA and Piwi-interacting RNA in genome defense (Landry et al., 2013; Ozata et al., 2019). RNA is present in cells and in extracellular vesicles which mediate signaling in-between cells and across tissues (Baldrich et al., 2019; O’Brien et al., 2020). It has also been causally involved in the transmission of phenotypes from parent to offspring in vertebrates and invertebrates (Krawetz, 2005; Bohacek and Mansuy, 2015; Rechavi and Lev, 2017).

Purification of high-quality RNA is an important step to investigate biological processes, cellular functions and phenotypes in molecular assays. The successful extraction of short and long RNA relies on the separation from DNA, proteins and cellular debris contained in cell lysate. Reverse transcription of purified RNA results in cDNA that can be amplified by polymerase chain reaction (PCR) for quantification of gene and transcript expression for classical qPCR or digital droplet PCR. Next generation sequencing is another method to quantify RNA, now commonly used to identify molecular signatures of tissues or individual cells and assess differential expression (Stark et al., 2019).

RNA can be purified from lysed cells by mainly two methods: acid guanidinium thiocyanate-phenol-chloroform (AGPC) and silica-based extraction columns (Chomczynski and Sacchi, 1987; Boom et al., 1990). AGPC extraction yields RNA of all lengths and is therefore the preferred method to obtain total RNA from cells and tissues (Toni et al., 2018). Its low cost, simplicity and adaptability to various biological material make it the most popular method in basic research. However, with this method, RNA purity and quality largely depend on the expertise of the experimenter and on sample handling. Silica-based columns allow nucleic acids extraction by binding to silica in the presence of chaotropic salts. It is commonly used in commercially available RNA extraction kits such as Qiagen RNAeasy and is amenable to automation for high throughput. Silica-based columns preferentially capture nucleic acids longer than 200 nucleotides but provide poor recovery of short RNA because short RNA tightly bind with silica and are less likely to elute (Ali et al., 2017). The recovered RNA is highly pure but the yield is usually lower when compared to AGPC.

In this study, we report that some cells, particularly mouse sperm cells, are resistant to commercially available AGPC and lysis solutions used for silica-based columns. Sperm cells cannot be properly lysed, which results in poor RNA yield and significant sample loss. Sperm lysis in AGPC is not improved by addition of the reducing agent β-mercaptoethanol (βME) or dithiothreitol (DTT), while βME added to the lysis solution in silica-based column protocols results in cell lysis. We observed that reduction of protein disulfide bonds is necessary for the lysis of sperm heads, and that reduction efficiency is pH-dependent, potentially explaining the difference in sperm lysis efficiency of various solutions. We identify tris(2-carboxyethyl)phosphine (TCEP) as a potent enhancer of AGPC RNA extraction and show that it allows complete lysis of mouse sperm cells.

## Materials and Methods

### Animals

Animal experiments were conducted in strict adherence to the Swiss Law for Animal Protection and were approved by the cantonal veterinary office in Zürich under license number 57/2015 and 83/2018. C57Bl/6J mice were obtained from Janvier (France) and bred in-house to generate male mice (*n* = 32, 5 months old) for experiments. Mice were housed in groups of 3–5 animals in individually ventilated cages. Animals were kept in a temperature- and humidity-controlled facility on a 12 h reversed light/dark cycle (light on at 20:00, off at 8:00) with food (M/R Haltung Extrudat, Provimi Kliba SA, Switzerland) and water *ad libitum*. Cages were changed weekly.

### Mouse Sperm Collection

Epididymidis from both sides was incised by several cuts with a fine scissor and placed in 2 ml M2 medium (M7167-100 ml, Sigma-Aldrich). Sperm cells were incubated at 37°C for 30 min. 1 ml of supernatant containing motile sperm was collected and centrifuged at 2,000 rcf for 5 min. The supernatant was again collected, mixed with 1 ml of somatic cell lysis buffer (0.1% sodium dodecyl sulfate and 0.05% Triton X-100 in MilliQ water) and incubated at 4°C for 10 min. Sperm samples were centrifuged at 2,000 rcf for 5 min, sperm pellets were washed twice in phosphate buffer saline (10010-015, Gibco) then snap-frozen and stored at −80°C until further processing. For lysis experiments, sperm samples were immediately resuspended in 250 μl water, lysis solutions were added and sperm observed under a light microscope.

### Cell Lysis

Sperm pellets and small pieces of testis, liver or epididymis were lysed in 1 ml of Trizol (15596026, Life Technologies) or 1 ml of Buffer RLT+ (1053393, Qiagen) depending on the experiments. Lysis buffer was supplemented with 100 mM β-mercaptoethanol (M3148, Sigma-Aldrich), 100 mM dithiothreitol (646563-10X.5ml, Sigma-Aldrich) or 100 mM tris(2-carboxyethyl)phosphine (646547-10 × 1ml, Sigma-Aldrich). Sperm samples were either resuspended in lysis buffer by passage through a 30 G syringe or in the presence of 0.2 mm steel beads (SSB02-RNA, NextAdvance) at 20 Hz for up to 10 min in a TissueLyser II (85300, Qiagen). Since the lysis of sperm cells depends primarily on chemical lysis, we recommend future users a simple homogenization to sufficiently break up the cell pellet to obtain a single-cell suspension using 5 mm steel beads for 2 min (69989, Qiagen). We did not observe any differences between sperm lysis using small or large steel beads. Tissue samples were homogenized in a TissueLyser II using 5 mm steel beads at 20 Hz for 2 min.

### RNA Extraction

For RNA extraction by phenol chloroform phase separation, 100 μl of sample lysed in Buffer RLT+ were added to 900 μl Trizol and extracted using the standard Trizol protocol. All centrifugation steps were at 4°C and samples kept on ice unless noted otherwise. In brief, 200 μl chloroform was added to 1 ml lysate, shaken for 15 s and then incubated at room temperature for 3 min. Samples were centrifuged at 12,000 rcf for 15 min. The aqueous phase was transferred to a fresh tube, 10 μl glycogen was added and the tube inverted 4 times for mixing. 500 μl isopropanol was then added and the tube inverted again 4 times. After an incubation at room temperature for 10 min, samples were centrifuged at 12,000 rcf for 10 min. The supernatant was removed and the pellet washed twice in 75% ethanol. After a final centrifugation at 7,500 rcf for 5 min, the supernatant was carefully removed. The pellet was dried in a concentrator (Speed-vac, Eppendorf) at 45°C for 4–6 min. Sperm RNA was resuspended in 20 μl nuclease-free water and tissue RNA in 100 μl nuclease-free water (A7398,500, ITW Reagents). Finally, the solution was incubated at 55°C for 15 min. RNA was stored at −80°C.

### RNA Quantification and Characterization

RNA concentration and integrity were analyzed on the 2100 Bioanalyzer (G2939BA, Agilent) with the RNA 6000 Pico Kit (5067-1513, Agilent) according to manufacturer instructions. DNA content was quantified by fluorometry with the Qubit double-strand DNA high sensitivity assay (Q32852, Invitrogen) according to manufacturer instructions. For RT-PCR of long RNAs, RNA samples were first DNase treated with the DNA-free Kit (AM1906, Invitrogen) and reverse-transcribed with the GoScript Reverse Transcription System (A5000, Promega) using random hexamers according to manufacturer instructions. A testis sample without GoScript reverse transcriptase (noRT) and a sample without RNA (no Input) was processed in parallel and served as negative controls. For qPCR of cDNA from long RNAs, 1 μl of cDNA per well was quantified using SYBR Green I Master (04887352001, Roche) and primers for β-actin (Forward (5′–3′): CGATGCCCTGAGGCTCTTTT, reverse (5′–3′): TAGAGGTCTTTACGGATGTCAACG). For end-point PCR of cDNA from long RNAs, 5 ng of cDNA was amplified by GoTaq G2 HS Polymerase (M7405, Promega) for 30 cycles using specific primers for Malat1 (Forward (5′–3′): ATCGATTTAAAGTAAATGGGCTA, reverse (5′–3′): TTACATGCAGGAACATTGACA) and Pgk2 (Forward (5′–3′): AAGTTTGATGAGAATGCTAAAGT, reverse (5′–3′): CCTCCTCCTATAATGGTGACA). PCR products size was assessed by agarose gel electrophoresis. In Figure 6F, a part of the gel between the DNA ladder and Malat1 PCR products was cut out because it contained an RT-PCR with a different primer set. For qPCR of miRNAs, RNA samples were reverse transcribed with miScript II RT reagents (218161, Qiagen) using HiFlex buffer. RT-qPCR was performed with QuantiTect SYBR Green PCR Kit (1046470, Qiagen) on a Light Cycler II 480 (Roche) using miScript Primers Assays (Qiagen) for miR-141 (MS00011165, Qiagen), miR-101b (MS00023919, Qiagen) and miR-200c (MS00032543, Qiagen). All samples were run in triplicate. Melt curve analysis confirmed amplification of single products for each primer.

### Quantification of Disulfide Bond Reduction

The reduction of disulfide bonds was performed as described previously (Han and Han, 1994). 1 mM 2,2′-dithiodipyridine (43791-1G, Sigma-Aldrich) was prepared in nuclease-free water and stored at 4°C. Lysis buffers were prepared as follows and used for absorption reference measurement: Trizol, Buffer RLT+, 1 M guanidinium thiocyanate (G9277-100g, Sigma-Aldrich) in nuclease-free water, 50 mM β-mercaptoethanol (βME) in nuclease-free water, Trizol supplemented with 50 mM βME (Trizol/βME), Buffer RLT+ supplemented with 50 mM βME (RLT+/βME) and Trizol supplemented with 50 mM TCEP (Trizol/TCEP). The reduction of DTDP was quantified by measuring the absorption of 2-thiopyridione (2-TPD) at 343 nm using an Ultrospec 2000 (80-2106-00, Pharmacia Biotech). 980 μl of lysis solution was prepared in UV-cuvettes and 20 μl of 1 mM DTDP solution added. The solution was briefly mixed by pipetting and absorption measurements started 10 s after DTDP addition. All experiments were repeated three times (*n* = 3).

### Data, Statistics and Visualization

Data are represented as mean ± standard error of mean (SEM). Two groups were compared by unpaired *t*-test (*p* < 0.05). For comparisons of qPCR data, groups were analyzed by ordinary one-way analysis of variance (ANOVA, *p* < 0.05). For comparisons in the DTDP assay, groups were analyzed by repeated measures one-way analysis of variance (ANOVA, *p* < 0.05). Significant effects in ANOVA were further analyzed by multiple comparison with Tukey’s *post hoc* test (adjusted *p*-value < 0.05). Graphs and statistics were prepared using the software GraphPad Prism 9. Contrast in microscopy pictures of Figure 5B was enhanced in Adobe PhotoShop 2021. Chemical reactions were drawn in ChemDraw19.

## Results

### Mouse Sperm Cells Are Resistant to AGPC RNA Extraction

**FIGURE 1:**
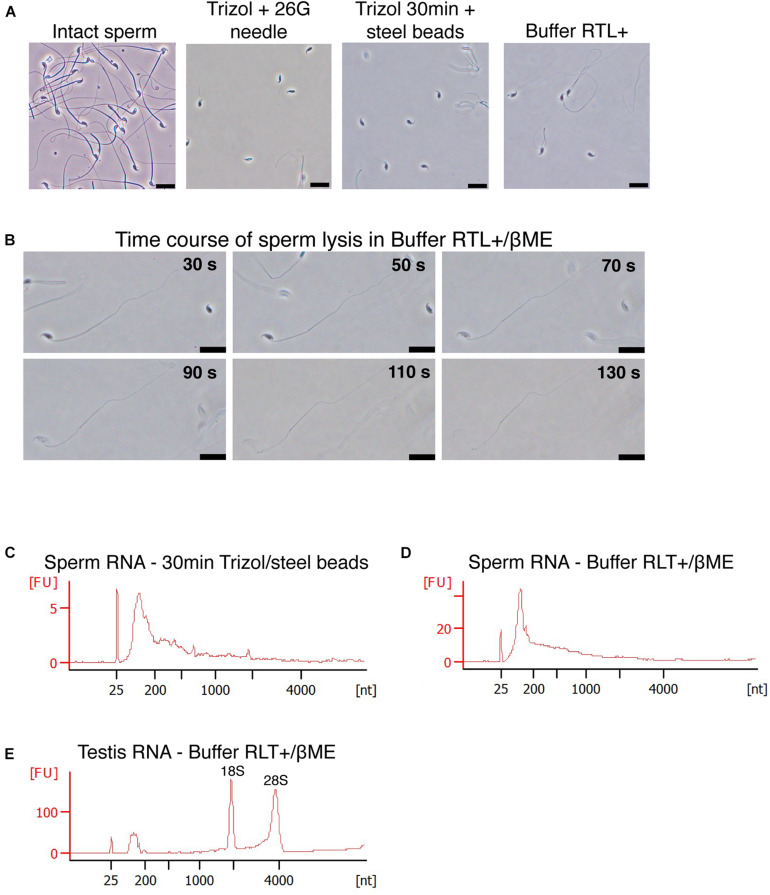
Mouse sperm cells require reducing agents for lysis in chaotropic solutions. **(A)** Mouse sperm is not lysed in Trizol nor Buffer RLT+. Lysis is not improved by mechanical shearing. Scale bars = 20 μm. **(B)** Mouse sperm is rapidly lysed by Buffer RLT+ supplemented with 50 mM βME. Scale bars = 20 μm. **(C–E)** Bioanalyzer electropherograms of RNA concentration in fluorescence units (FU) for a given nucleotide length (nt). Sperm RNA prepared with **(C)** Trizol and homogenized by steel beads, or **(D)** Buffer RLT+ and βME, and **(E)** testes RNA prepared with Buffer RLT+ and βME.

A sperm cell is composed of a head containing a nucleus that carries the paternal genome and RNA, and a flagellum prolonging the head through a mitochondria-rich midpiece, that provides motility (Figure 1A). We incubated mouse sperm cells at room temperature in Trizol, a commercially available AGPC RNA extraction reagent containing guanidinium thiocyanate as chaotropic agent. To separate sperm heads from the midpiece and flagellum, and dissociate cell clumps, we passed the sperm samples 10 times through a 30 G needle at the beginning of Trizol incubation. Despite this treatment, sperm heads remained intact after 5 min of incubation, indicating inefficient lysis (Figure 1A). We repeated the procedure and made it more stringent by using longer incubation (30 min) in Trizol and strong homogenization with small steel beads at 20 Hz for 10 min. However, intact sperm heads still remained visible (Figure 1A). These results confirm previous observations that 2 M guanidinium does not lyse sperm heads (Flaherty and Breed, 1983).

We examined if other commercially available RNA extraction solutions are more efficient. Buffer RLT+ is a proprietary lysis solution of the Qiagen RNAeasy Mini kit, whose composition is unknown. However, the material safety data sheet suggests that it contains up to 50% guanidinium thiocyanate, thus resembles chaotropic properties of Trizol. Buffer RLT+ did not allow sperm lysis either (Figure 1A) even with a prolonged incubation of 18 h at room temperature. However, complete lysis of sperm heads was achieved within 2 min when 100 mM β-mercaptoethanol (βME) was added to Buffer RLT+ at room temperature (Figure 1B). βME is a commonly used reducing agent that breaks inter- and intradisulfide bonds formed between cysteine residues in proteins. The addition of βME is recommended by the manufacturer of the RNAeasy Mini kit to inhibit RNAse, especially when extracting RNA from pancreas and spleen, tissues rich in RNAse.

We extracted RNA from Trizol and Buffer RLT+/βME sperm lysates using a standard phenol-chloroform protocol. To exclude contamination by testicular somatic cells, we prepared sperm samples using a swim-up method followed by treatment with a somatic cell lysis buffer. This approach has previously been shown to yield sperm samples free of somatic cells (Peng et al., 2012; Bianchi et al., 2018). We observed cell debris but no discernible somatic cells in the samples before RNA extraction. We assessed the quality of the extracted RNA by gel electrophoresis. Sperm samples were free of distinct 18S/28S rRNA peaks and had the expected RNA profile (Figures 1C,D), whereas RNA from testes had two characteristic peaks at 1,800 and 3,800 nt for 18S and 28S ribosomal RNA, respectively, and are apparent that indicate high-quality RNA (Figure 1E), suggesting no contamination by somatic cells.

These results indicate that the standard protocol for AGPC RNA extraction is inappropriate to completely lyse mouse sperm cells and is the main reason for poor RNA yield from these cells, and that using Buffer RLT+/βME instead of AGPC solutions circumvents this issue.

### Examining Sperm Lysis Properties and Reduction of Disulfide Bonds

**FIGURE 2:**
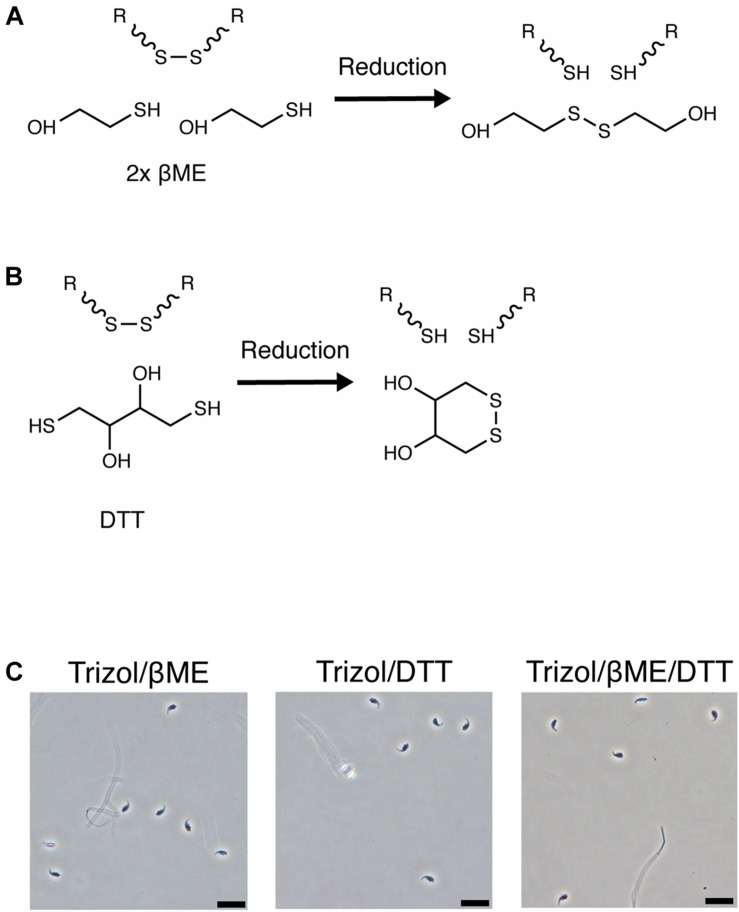
Sperm is not lysed in Trizol supplemented with βME or DTT. **(A,B)** Chemical reduction of a protein disulfide bond by **(A)** βME or **(B)** DTT. **(C)** Mouse sperm is not lysed in Trizol supplemented with 100 mM βME, 100 mM DTT or a combination of both. Scale bars = 20 μm.

We next examined the reason for the lysis-resistance of sperm outer membrane. Sperm cells are lysed by Buffer RLT+ only if supplemented with βME despite the presence of guanidinium thiocyanate as main chaotropic agent. βME is a widely used reducing agent that disrupts disulfide bonds between thiol groups such as cysteine residues, within and between proteins (Figure 2A). During RNA extraction, it inhibits ribonuclease activity and prevents the degradation of RNA released in solution. We tested if adding a reducing agent to AGPC solution results in the successful lysis of sperm cells. Trizol with 100 mM βME however did not lead to the lysis of sperm heads, which remained intact (Figure 2C). Similarly, Trizol supplemented with dithiothreitol (DTT, Figure 2B) did not induce lysis (Figure 2C) even if DTT has been shown to decondense sperm heads by reducing disulfide bonds in protamines (Flaherty and Breed, 1983; Zirkin et al., 1989).

We then examined if the pH of the lysis solution influences the efficiency of sperm lysis. The pH primarily affects the reactivity of reducing agents and an alkaline pH is necessary for efficient reduction of disulfide bonds by βME and DTT (Han and Han, 1994). In contrast, an acidic pH is preferred for protein disulfide bond mapping by mass spectrometry because it preserves disulfide bonds (Lakbub et al., 2018). We measured the pH of several commercially available RNA extraction solutions and observed that Trizol and other AGPC lysis buffers have a pH of 3 while guanidinium thiocyanate in water has a pH of 5 and Buffer RLT+ and lysis buffer from mirVana RNA extraction kit using silica-columns have a pH of 6 (Table 1). The low pH may explain why βME did not improve lysis by Trizol.

**TABLE 1:** pH of commercially available lysis solutions for RNA extraction.

Commercial name	RNA extraction method	pH
TRIzol reagent	AGPC	3
QIAzol	AGPC	3
Tri reagent	AGPC	3
TRIsure	AGPC	3
Guanidinium thiocyanate 1 M	AGPC	5
Buffer RLT+	Silica columns	6
mirVana lysis/binding buffer	Silica columns and AGPC	6

**FIGURE 3:**
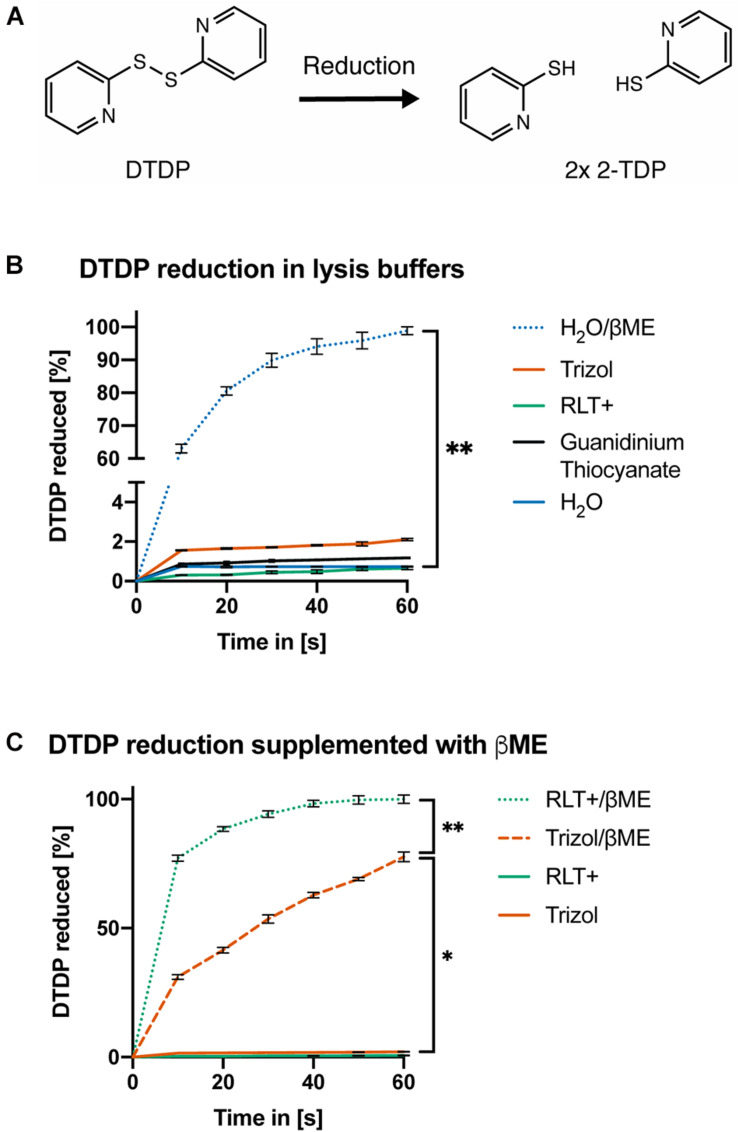
DTDP assay reveals pH-dependent activity of reducing agents. **(A)** Reduction of 2,2′-Dithiodipyridine (DTDP) produces 2-thiopyridione (2-TPD). **(B,C)** Measurement of DTDP reduction in % during 60 s. **(B)** DTDP is not reduced by Trizol, Buffer RLT+, 1 M guanidinium thiocyanate or water. Water supplemented with 50 mM βME reduces DTDP within 60 s. **(C)** Buffer RLT+ supplemented with βME completely reduces DTDP. Trizol supplemented with βME reduces 75% of DTDP in the same time. Each condition was repeated 3 times (*n* = 3). Data are represented as mean ± standard error of mean (SEM). Significant ANOVAs were followed by Tukey’s *post hoc* test, *p < 0.05, **p < 0.01.

Finally, we confirmed the pH-dependent activity of reducing agents by measuring the amount of 2-thiopyridione (2-TPD) formed after reduction of 2,2′-dithiodipyridine (DTDP) in lysis solutions. DTDP mimics protein disulfide bonds between cysteines and absorbance at 343 nm of 2-TPD in solution indicates the amount of disrupted bonds (Figure 3A). Trizol, Buffer RLT+ or 1 M guanidinium thiocyanate did not reduce DTDP. However, Buffer RLT+ supplemented with 50 mM βME led to rapid (within 40 s) and complete reduction of DTDP in water [Figure 3B, ANOVA, *F*(1.000, 6.000) = 31.6, *p* = 0.0014]. When added to Trizol, 50 mM βME led to only slow and incomplete reduction of DTDP, with 25% remaining after 60 s [Figure 3C, ANOVA, *F*(1.171, 7.026) = 29.94, *p* = 0.0007].

In summary, the supplementation of acidic AGPC solutions with βME or DTT is not sufficient to lyse mouse sperm, likely because guanidinium thiocyanate cannot reduce disulfide bonds present in the sperm head membrane, and the reactivity of βME is pH dependent.

### TCEP Enhances AGPC Lysis

**FIGURE 4:**
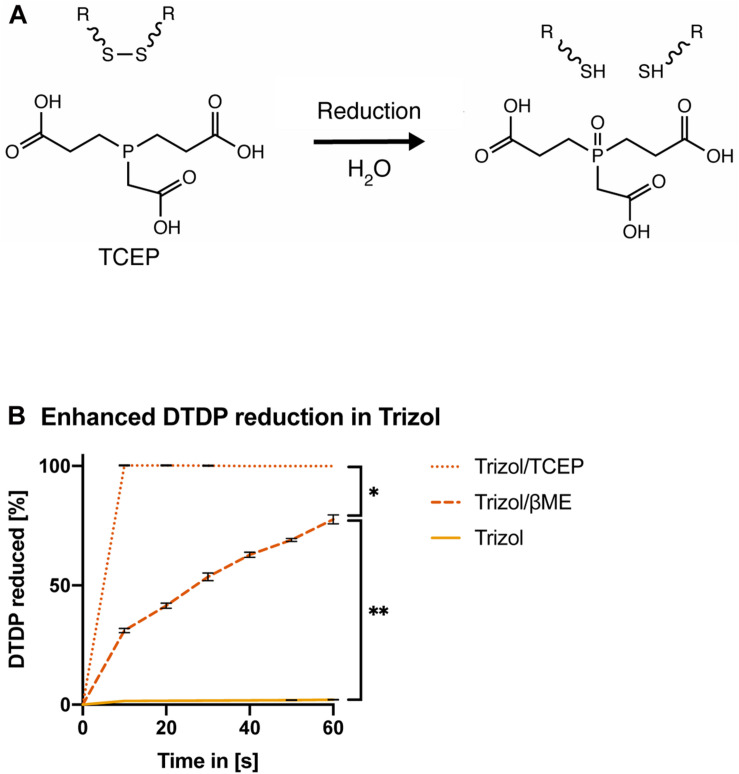
TCEP is a potent reducing agent in AGPC lysis solution. **(A)** Chemical reduction of a protein disulfide bond by TCEP. **(B)** Measurement of DTDP reduction in % during 60 s. DTDP was rapidly reduced in Trizol supplemented with 50 mM TCEP within 10 s. Each condition was repeated 3 times (*n* = 3). Data are represented as mean ± standard error of mean (SEM). Significant ANOVAs were followed by Tukey’s *post hoc* test, *p < 0.05, **p < 0.01.

Tris(2-carboxyethyl)phosphine (TCEP) was previously shown to effectively reduce disulfide bonds across a wide pH range from 1.5 to 8.5 (Figure 4A; Han and Han, 1994). Therefore, we first assessed if TCEP could be an effective reducing agent for acidic lysis solutions. In contrast to βME, Trizol supplement with 50 mM TCEP rapidly reduced all DTDP within 10 s [Figure 4B, ANOVA, *F*(1.458, 8.747) = 28.96, *p* = 0.0002].

**FIGURE 5:**
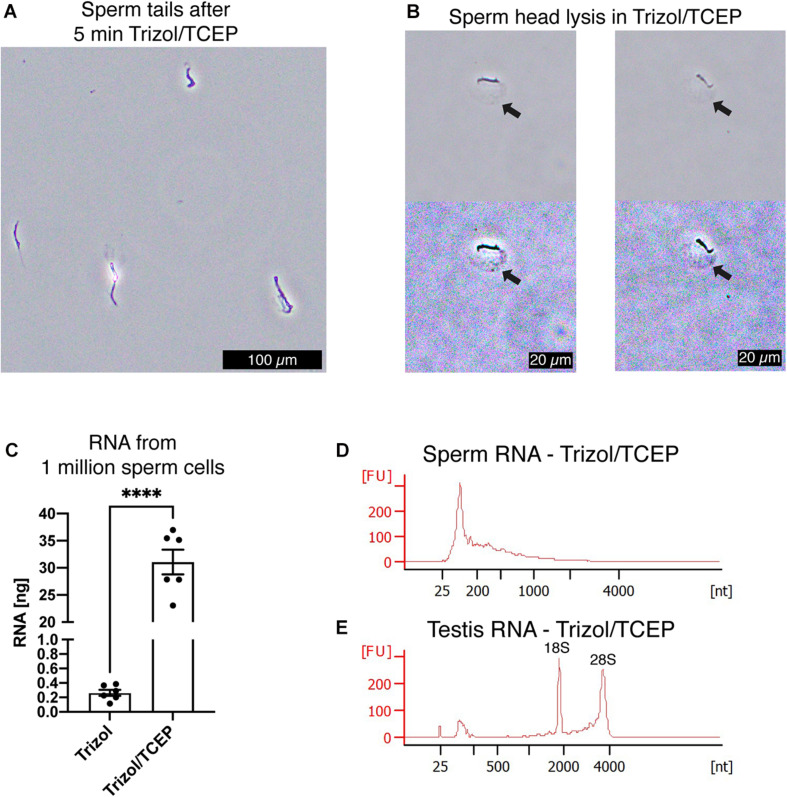
TCEP enhances AGPC lysis and increases sperm RNA yield. **(A)** Mouse sperm cells are rapidly lysed in Trizol supplemented with 50 mM TCEP. Only clumped sperm tails remain visible indicating complete lysis of sperm heads. Scale bar = 100 μm. **(B)** Details of mouse sperm lysis in Trizol/TCEP. In comparison to lysis of mouse sperm in Buffer RLT+/βME, the acrosome remains visible for longer and a distinct swelling of the nucleus containing sperm head is observed (black arrows). In the second row, color contrast of microscopy pictures was increased to highlight the swelling. Scale bars = 20 μm. **(C)** Comparison between RNA yield in ng from 1 millions sperm cells when extracted with only Trizol or Trizol supplemented with TCEP. Trizol/TCEP extraction increased the RNA yield to an average of 30 ng (*n* = 6 per lysis condition). **(D,E)** Bioanalyzer electropherograms of RNA concentration in fluorescence units (FU) for a given nucleotide length (nt). **(D)** Sperm RNAs extracted from Trizol/TCEP were comprised mostly of RNAs shorter than 200 nt. **(E)** Testis RNA extracted from Trizol/TCEP was comprised of longer RNAs with distinct peaks at 1,800 and 3,800 nt. Data are represented as mean ± standard error of mean (SEM). ****p < 0.0001.

Next, we examined if lysis of sperm cells was improved by supplementing AGPC lysis solutions with TCEP. We observed complete lysis of all sperm heads in Trizol with 50 mM TCEP at room temperature within 5 min (Figures 5A,B). As expected, complete sperm lysis improved the efficiency of RNA extraction without requiring any adjustment or additional recovery step of the standard AGPC protocol. 1 million sperm cells yielded 30 ng RNA when lysed by Trizol/TCEP, thereby considerably improving RNA yield (Figure 5C). Sperm RNA profile had no distinct 18S/28S ribosomal peaks indicating that the source of increased RNA was from sperm cells rather than contamination by somatic cells (Figure 5D). Similarly to Buffer RTL+/βME, extraction by Trizol/TCEP from testis tissue yielded high-quality RNA (Figure 5E). We observed the likely presence of genomic DNA in the RNA profiles of a sperm sample (Supplementary Figure 1A). Subsequent DNA quantification by fluorometry showed the presence of genomic DNA in all sperm RNA samples [Supplementary Figure 1B, ANOVA, *F*(3, 12) = 18.55, *p* < 0.0001]. Therefore, all samples were DNAse treated before further use. To exclude RNA degradation, somatic RNA samples were processed in parallel and no substantial decrease in RNA integrity values was observed (Supplementary Figure 1C).

**FIGURE 6:**
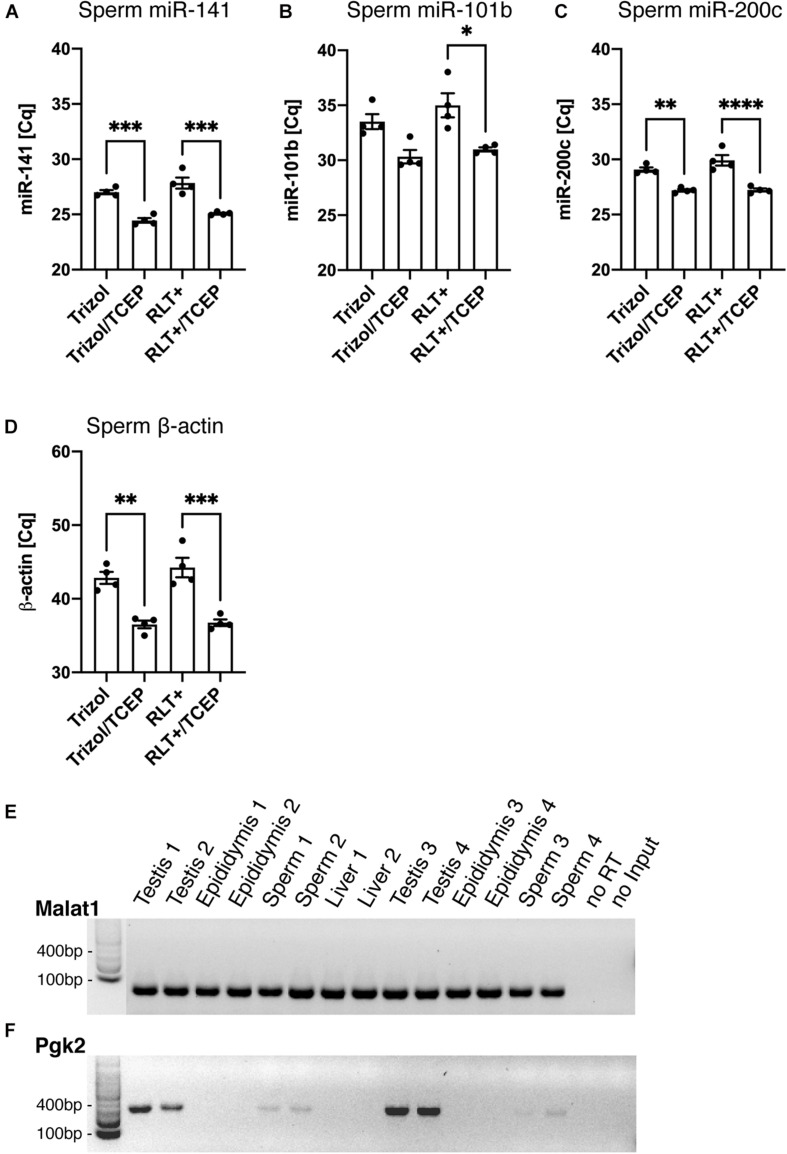
Improved RNA yield quantified by qPCR of specific RNAs. **(A–D)** Quantification of RNA amount by qPCR. Detected cycle thresholds (Cq) are plotted on the y-axis and lower Cq values signify more starting material. **(A–C)** qPCR of miRNAs miR-141, -101b, and -220c. (*n* = 4 per group). **(D)** qPCR of long RNA β-actin. (*n* = 4 per group). **(E,F)** Gel images of end-point RT-PCR. **(E)** lncRNA Malat1 is detected by RT-PCR in Trizol/TCEP samples from sperm (*n* = 4), testis (*n* = 4), epididymis (*n* = 4) and liver (*n* = 2). **(F)** The germ-line specific transcript Pgk2 is only present in sperm and testis. noRT = RNA not reverse transcribed. NoInput = Sample without RNA. Significant ANOVAs were followed by Tukey’s *post hoc* test, *p < 0.05, **p < 0.01, ***p < 0.001, ****p < 0.0001.

Finally, we confirmed that the RNA extracted by Trizol and TCEP was suitable for downstream applications such as reverse transcription polymerase chain reaction (RT-PCR). To determine the differences in RNA yield by quantitative PCR (qPCR), all sperm samples were processed similarly from lysis to final reverse transcription without any adjustment to volumes at the different processing steps. This ensured that initial differences in RNA quantity remained until qPCR and were quantified by reporting the cycle threshold number (Cq). First, we conducted qPCR on miR-141 [Figure 6A, ANOVA, *F*(3, 12) = 30.42, *p* < 0.0001], miR-101b [Figure 6B, ANOVA, *F*(3, 12) = 9.15, *p* = 0.002], and miR-200c [Figure 6C, ANOVA, *F*(3, 12) = 25.34, *p* < 0.0001]. Lower cycle values in qPCR indicate faster amplification and therefore higher RNA concentration in the sample. We observed significantly faster amplification of RNA samples obtained from lysis in Trizol/TCEP and RLT+/TCEP when compared to incomplete lysis (Figures 6A–C).

Next, we conducted qPCR on the reference gene β-actin and detected significantly faster amplification in sperm RNA samples obtained from lysis with Trizol/TCEP and RLT+/TCEP [Figure 6D, ANOVA, *F*(3, 12) = 22.6, *p* < 0.0001]. Furthermore, we conducted end-point PCR for two less abundant genes, metastasis associated lung adenocarcinoma transcript 1 (Malat1) and germ-cell specific phosphoglycerate kinase 2 (Pgk2). Malat1, a long non-coding RNA expressed in many mouse tissues (Hutchinson et al., 2007), amplified in sperm, testis, epididymis and liver RNA samples extracted by Trizol/TCEP (Figure 6E). In contrast, Pgk2 only amplified in sperm and testis samples (Figure 6F). In addition, we performed qPCR for Malat1 and Pgk2. However, all sperm samples amplified above 40 cycles and were considered as not detectable (data not shown; Bustin et al., 2009).

In conclusion, the supplementation of AGPC with TCEP enhances the lysis and RNA extraction from sperm cells, and considerably increases the RNA yield from these cells.

## Discussion

This study shows that mouse sperm cells are resistant to RNA extraction by AGPC and that supplementation with TCEP solves this issue by allowing complete lysis and high RNA yield. The results indicate that a disulfide reducing agent is necessary to lyse sperm cells, suggesting that disulfide bonds confer chemical resistance to sperm cells, in mouse and potentially other species.

The efficiency of AGPC extraction depends on the complete lysis of cells in the presence of chaotropic guanidinium (Gdm^+^) and thiocyanate (SCN^–^) ions, and additional factors such as mechanical shearing, degrading conditions and osmotic pressure. Gdm^+^ and SCN^–^ ions are strong protein denaturants that partially disrupt accessible hydrophilic bonds, and are commonly used in studies of protein stability and folding (Baldwin, 1996; Mason et al., 2003). They also strongly inhibit the enzymatic activity of ribonucleases released during cell lysis, thus prevent the degradation of RNA in solution and contribute to the acidification of the lysate (Chang and Li, 2002). An acidic pH is necessary during phenol-chloroform separation where DNA and proteins move to the organic phase while RNA remains in the aqueous phase (Chomczynski and Sacchi, 1987, 2006).

Despite the general applicability and potency of AGPC lysis solutions, we observed that mouse sperm cells are not effectively lysed and require the presence of pH-compatible denaturing agents. Specifically, we show that TCEP improves lysis at acidic pH. TCEP is also used to stabilize extracted RNA, for the reduction of proteomic samples and the preparation of protein:RNA crystallization samples (Rhee and Burke, 2004; Ripin et al., 2019; Thomas et al., 2020).

Sperm cells are specialized cells whose functions are to maintain the paternal genome and transfer it to an oocyte to generate offspring. During the last stages of spermatogenesis, most histones are replaced by protamines in sperm and only 1–8% are retained in mouse and 10–15% in human. Histones are retained principally at genomic regions such as transcription start sites, promoters and enhancers (Jung et al., 2017; Yamaguchi et al., 2018; Lismer et al., 2020). Protamines have different properties to histones, and in contrast to histones, they are highly enriched in disulfide bonds. Intra- and inter-protamine bonds mediate the packaging of DNA into a crystalline-like toroid and stabilize this structure, which requires reducing agents for its decondensation (Hisano et al., 2013; Hutchison et al., 2017). The highly condensed sperm nucleus is surrounded by the acrosome which in mice, has a distinct hook-like shape for cooperative cell motility, and carries enzymes necessary for successful sperm-oocyte fusion (Moore et al., 2002). The acrosome protects the sperm nucleus from the environment of the vagina and uterus during transit to the oviduct. It is primarily composed of proteins, ceramides and sphingomyelins with very long chain (carbon length 24–34) polyunsaturated fatty acids (PUFA) (Robinson et al., 1992). The acrosome composition varies between mouse, rat and human with predominantly very long chain PUFA (C30) in mouse, (C28) in rat and (C16) in human (Zanetti et al., 2010; Craig et al., 2019). Molecular dynamics simulations have shown that the composition regulates the stability and mechanical rigidity of the bilayer membrane, and changes in very long chain PUFA composition have been implicated in reduced sperm quality (Ahumada-Gutierrez et al., 2019; Craig et al., 2019). Our findings suggest a critical role for disulfide bonds in conveying chemical resistance of sperm cells, which has not been described previously.

The resistance of mouse sperm cells to acidic pH may be a form of cellular adaptation to protect sperm cells during transit in the vagina and uterus. While the testicular environment and epididymis are chemically stable in male mice, the uterus and vaginal milieu in females undergo substantial changes during the estrous cycle. The mouse vagina has typically a slightly acidic pH to suppress the growth of undesired bacteria and fungi. Importantly, the acidic environment together with other secreted enzymes contributes to the capacitation of sperm cells. The vaginal pH fluctuates with the estrous cycle and is particularly acidic (pH 4.5) during the estrous phase (Ganesan and Kadalmani, 2016). This is a time when females are the most receptive for copulation (Byers et al., 2012), thus the sperm is naturally exposed to an acidic environment. Its ability to resist such environment therefore allows it to regulate intracellular pH and maintain physiologically relevant functions (Nishigaki et al., 2014).

Since sperm cells are transcriptionally silent, a low RNA yield is expected from these cells. In sperm cells, protein synthesis is suppressed by cleavage of ribosomal RNA and most of the RNA in the cytoplasm is expulsed during spermatogenesis. A single sperm cell likely contains only 10–100 fg RNA (Pessot et al., 1989). Thus, an efficient method for RNA extraction is essential to recover sufficient RNA for RT-PCR and sequencing to avoid pooling several sperm samples. Furthermore, low RNA yield can also confound downstream analyses because RNA species such as miRNAs can be selectively lost during AGPC RNA extraction when using a small number of cells (Kim et al., 2012). With the improved lysis of sperm heads, we obtained on average 30 fg RNA per sperm cell, consistent with previous studies (Krawetz, 2005). This was sufficient to perform qPCR for the quantification of the miRNAs miR-141, -101b, and −200c but long RNA targets such as β-actin, Malat1, and Pgk2 required more amplification cycles. This difference in RNA abundance should be taken into account when selecting targets for RNA quantification in sperm.

While we focused on the lysis of mouse sperm cells, similar issues and resistance to AGPC or other chaotropic solutions have been reported for sperm from other species such as human (Bianchi et al., 2018), bovine (Gilbert et al., 2007) and chicken (Shafeeque et al., 2014), and for extracellular vesicles (Tang et al., 2017) and non-enveloped viruses (Schlegel et al., 2001). Further to RNA, other macromolecules such as DNA, proteins or metabolites will also benefit from the improved lysis method. Further, with the availability of single cell sequencing, the reliable lysis and characterization of each cell becomes even more important. For example, easy to implement protocols such as genome and transcriptome sequencing (G&T-seq) allow parallel sequencing of genomic DNA and RNA from single cells (Macaulay et al., 2015). However, G&T-seq uses Buffer RLT+ without any reducing agent to lyse cells. Supplementing Buffer RLT+ with TCEP may lead to faster and more robust cell lysis. Therefore, our findings can benefit other model organisms and single-cell applications requiring efficient cell lysis.

## Data Availability Statement

The original contributions presented in the study are included in the article, further inquiries can be directed to the corresponding author.

## Ethics Statement

The animal study was reviewed and approved by cantonal veterinary office in Zürich.

## Author Contributions

MR designed, performed, analyzed and interpreted all experimental work. IM supervised and acquired funding for the study. MR and IM wrote the manuscript. Both authors discussed the results and approved the final version of the manuscript.

## Conflict of Interest

The authors declare that the research was conducted in the absence of any commercial or financial relationships that could be construed as a potential conflict of interest.

## Supplementary Material

Supplementary Figure 1 Quantification of residual genomic DNA in sperm RNA samples. **(A,C)** Bioanalyzer electropherograms of RNA concentration in fluorescence units (FU) for a given nucleotide length (nt). **(A)** Unusual peak at 2,000 nt is residual genomic DNA. Subsequent treatment with DNAse removes the peak. **(B)** Quantification of residual genomic DNA by fluorometry and plotted as a ratio against RNA amount per sample. (n = 4 per group). **(C)** Quantification of RNA integrity in somatic RNA shows no decrease of RNA integrity after DNAse treatment. Significant ANOVAs were followed by Tukey’s *post hoc* test, ^∗^*p* < 0.05, ^∗∗^*p* < 0.01, ^∗∗∗^*p* < 0.001.

## Abbreviations

β ME: β -mercaptoethanol
RNA: ribonucleic acid
2-TDP: 2-thiopyridione
AGPC: Guanidinium thiocyanate-phenol-chloroform
PUFA: polyunsaturated fatty acids
TCEP: Tris(2-carboxyethyl)phosphine
DTDP: 2,2 ′ -dithiodipyridine
RT-PCR: reverse transcription polymerase chain reaction
DTT: dithiothreitol
G&T-seq: genome and transcriptome sequencing
ANOVA: analysis of variance.
