# Supplementary figures and images for: High Efficiency RNA Extraction From Sperm Cells Using Guanidinium Thiocyanate Supplemented With Tris(2-Carboxyethyl)Phosphine
Source: Front Cell Dev Biol. 2021 Apr 21;9:648274. doi: 10.3389/fcell.2021.648274 (PMC8097045; doi:10.3389/fcell.2021.648274)

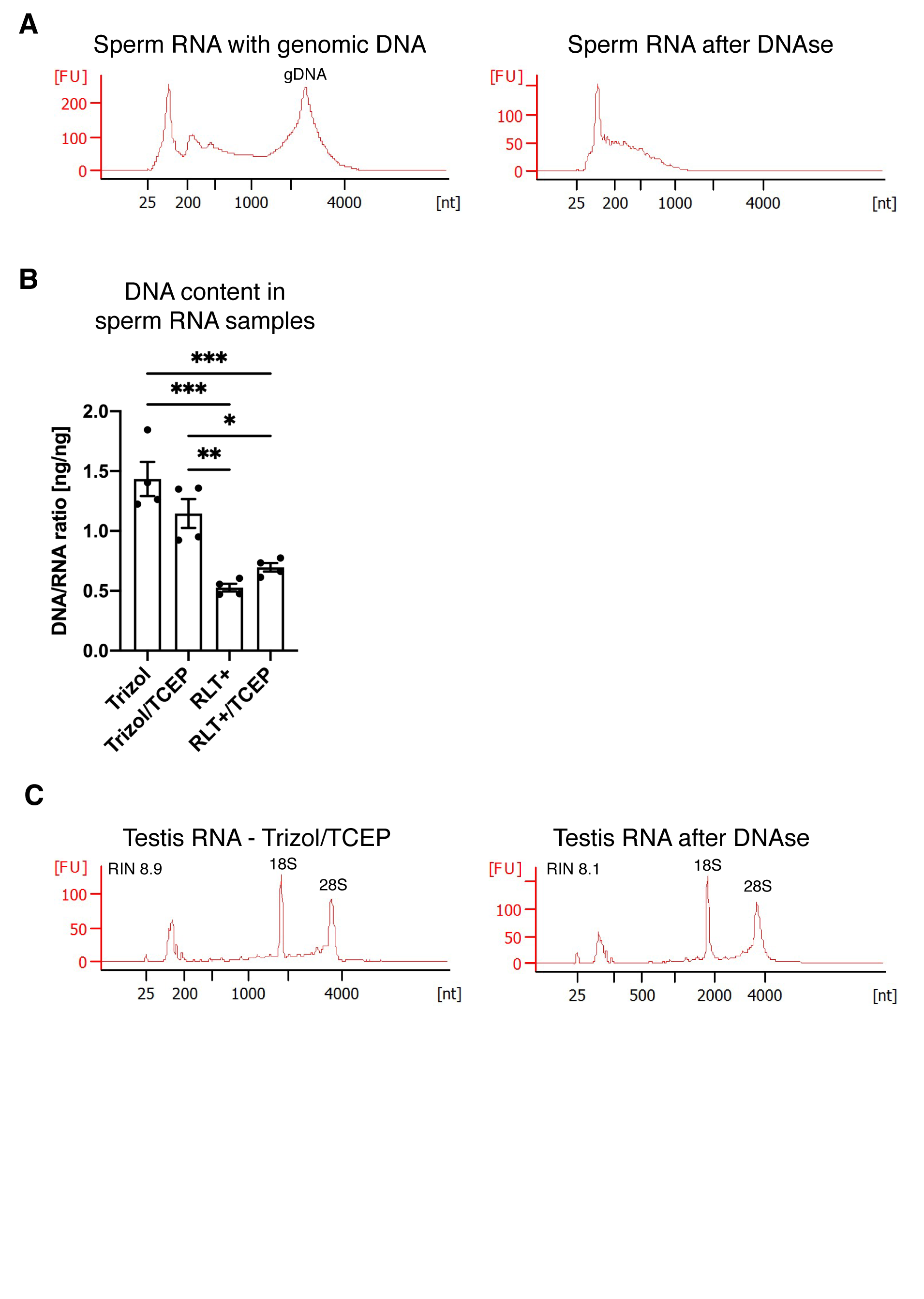

Supplement: Supplementary Figure 1 — Quantification of residual genomic DNA in sperm RNA samples. (A,C) Bioanalyzer electropherograms of RNA concentration in fluorescence units (FU) for a given nucleotide length (nt). (A) Unusual peak at 2,000 nt is residual genomic DNA. Subsequent treatment with DNAse removes the peak. (B) Quantification of residual genomic DNA by fluorometry and plotted as a ratio against RNA amount per sample. (n = 4 per group). (C) Quantification of RNA integrity in somatic RNA shows no decrease of RNA integrity after DNAse treatment. Significant ANOVAs were followed by Tukey’s post hoc test, ∗p < 0.05, ∗∗p < 0.01, ∗∗∗p < 0.001. [file Image_1.TIF]
